# Addressing Patient–Provider Communication Gaps in Vanishing Twin Syndrome: Implications for Patient Care and Clinical Guidelines

**DOI:** 10.3390/healthcare13162048

**Published:** 2025-08-19

**Authors:** Nichole M. Cubbage, Samantha L. P. Schilit, Allison Groff, Stephanie Ernst, Marc A. Nascarella

**Affiliations:** 1 Department of Public Health, Behavioral and Health Sciences, Massachusetts College of Pharmacy and Health Sciences, Boston, MA 02115, USA; marc.nascarella@mcphs.edu; 2 Myriad Genetic Laboratories, Inc., Salt Lake City, UT 84116, USA; samanthaschilit@gmail.com; 3 Capital Women’s Care, Frederick, MD 21702, USA; agroff@cwcare.net; 4 TAPS Support Foundation, 1326 HS Almere, The Netherlands; stephanie@tapssupport.com

**Keywords:** vanishing twin syndrome, patient–provider communication, bereavement support, clinical information, multiple pregnancies, twin pregnancies, fetal loss, miscarriage

## Abstract

**Background**: Vanishing twin syndrome (VTS) represents a complex and under-recognized phenomenon in multifetal pregnancies, associated with both clinical uncertainty and significant psychosocial impact. Despite its frequency, gaps remain in diagnostic clarity, international guidelines, and communication strategies with patients and families. **Materials and Methods**: This hybrid review integrates narrative and systematic elements to assess the diagnostic, clinical, and psychosocial gaps in VTS. A systematic literature search was conducted across Medline/PubMed, CINAHL, PsycINFO, EBM Reviews, and Scopus using terms such as “vanishing twin syndrome,” “patient-provider communicat*,” and “bereave* care.” Sources included systematic reviews, randomized controlled trials, cohort studies, and qualitative studies. Exclusion criteria were outdated publications (>10 years old). **Results**: Evidence revealed multiple domains of concern. Clinical risks and diagnostics remain poorly defined, with inconsistent recognition of maternal and neonatal complications. Psychosocial impacts were prominent, encompassing grief, identity disruption, and unmet support needs. Patient–provider communication was frequently inadequate, with insufficient training and lack of standardized language. International guidelines varied widely in scope, with only a few of them providing clear recommendations for bereavement care in multifetal loss contexts. **Discussion**: Emerging discourse highlights the limitations of the traditional fission model and alternative conceptual frameworks, such as Herranz’s model, for understanding VTS. These theoretical differences underscore the need for precise terminology and consistent diagnostic practices. Clinical implications extend to prenatal screening, obstetric management, and the integration of psychosocial support. Patient-centered communication and structured support initiatives (e.g., the Butterfly Project) demonstrate the potential to bridge communication gaps and improve care experiences. **Conclusions**: VTS requires recognition as both a medical and psychosocial condition. Improved clinical definitions, harmonized international guidelines, and emphasis on empathetic communication are essential to address the current gaps. Integrating these elements into practice may enhance patient outcomes and provide families with validation and support following multifetal loss.

## 1. Introduction

Vanishing twin syndrome (VTS) refers to the occurrence where a twin or multiple gestation pregnancy is diagnosed, but one or more embryos or fetuses die or cease to develop during any trimester in gestation [1]. However, this definition is controversial, as some researchers define VTS exclusively as a first-trimester event, whereas others acknowledge that fetal losses meeting similar clinical criteria may also occur later in gestation [1,2]. The literature has also presented the term “vanishing twins syndrome” (where twins is plural) and consequently solely analyzed twin pregnancies where both sacs or embryos disappeared [3]. In both terms, complete vanishment may be implied misleadingly, and the plural version may further imply falsely that more than one twin vanishes. From some definitions, VTS is exclusively applicable to twin pregnancies and not higher-order multiples, reflecting another layer of complexity with the term [4]. VTS may result in one of the following outcomes, which vary by the stage of development at the time of loss, cause of death, and type of multiples:

### 1.1. The Three General Types of VTS Outcomes

1. Complete Resorption

This outcome typically occurs in the first trimester. The deceased gestational sac, embryo, or fetus may be completely or partially resorbed into the pregnant individual and/or the surviving co-twin(s). Biological materials such as fetal cells, RNA/DNA, proteins, and hormones may enter the maternal circulation and/or integrate into the co-twin(s) [1]. These losses occur slightly later than those involving a blighted ovum.

2. Retention of Remnants

In some cases, fetal or gestational remnants persist throughout pregnancy and, similar to cases of full resorption, biological material may enter the maternal circulation and/or integrate into the co-twin(s) [1]. This includes:

- Blighted ovum (anembryonic pregnancy): A gestational sac forms without an embryo. Diagnosed typically at 6–7 weeks, it often resorbs by the end of the first trimester but can persist throughout pregnancy in some cases [1,5,6].
- Residual embryonic, fetal, and placental remains: Tissues from a demised conceptus may remain visible on ultrasound or at birth and may contribute to cell-free DNA (cfDNA) or protein in the maternal bloodstream throughout gestation [1].

3. Fusion or Integration (Including Mummification)

**Figure 1:**
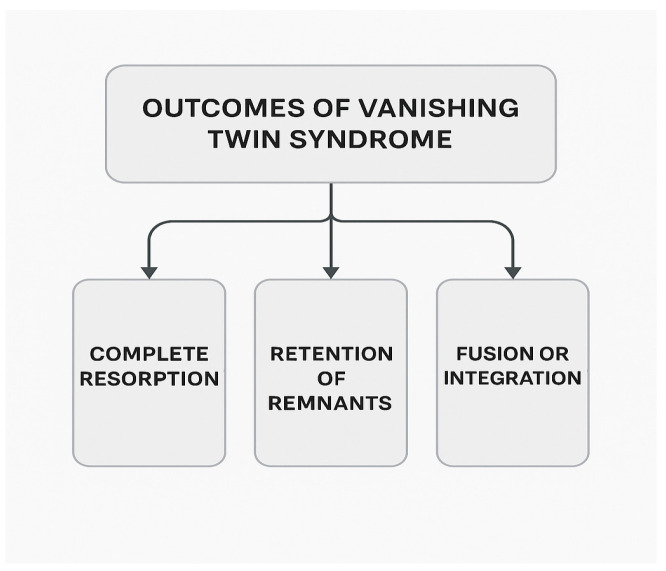
General VTS outcomes.

Later gestational losses may result in more complex integration of fetal tissue. For example, fetus papyraceous, which is a rare but severe form of VTS where the deceased fetus becomes mummified and compressed against the uterine wall or membranes. This typically occurs in the second or third trimester and is almost always visible at birth. It poses significant risks to the pregnant individual and surviving multiples [1]. Figure 1 below depicts the three general outcomes of VTS:

### 1.2. History and Recognition of VTS

The concept of vanishing twin was first described in the medical literature in the 1940s by a German obstetrician, Walter Stoeckel, who noted that the rate of multiple pregnancies was far greater than that of their births [7]. However, the term “vanishing twin syndrome” was not first coined until presented in a literature review [2]. Since that time, VTS has become more widely recognized due to advancements in ultrasound technology [1]. Twinning rates and subsequent rates of VTS have increased both with increasing use of assisted reproductive technologies (ART) (i.e., due to multiple embryo transfer or hyperovulation) and due to rising maternal age, yet research on VTS has lagged due to historical underdiagnosis of VTS [1,3]. The absence of significant literature on the subject has led to inconsistent patient–provider communication, inadequate standardized care protocols, and significant emotional distress for affected families. This systematic review synthesizes existing research on VTS, including its impact on the current state of patient–provider communication, interpretation of prenatal screening results, fetal development, maternal health, and the challenges in standardizing medical protocols.

### 1.3. Inadequate Diagnosis of VTS

While some individuals with VTS could be diagnosed postpartum (e.g., fetus papyraceous), VTS patients as a population were, by technological and diagnostic limitations, under-recognized before ultrasound was used widely in obstetric and gynecological medicine in the 1970s–1980s in most developed nations [8]. VTS is frequently underreported, particularly in naturally conceived pregnancies, as most cases “vanish” (i.e., resorb) completely within the first trimester—often before a patient’s first prenatal appointment, which typically occurs between 8 and 13 weeks gestation [1]. Even when prenatal care begins early, cases of VTS may still go unrecorded due to inadequate diagnostic terminology and limited provider awareness of the syndrome. For instance, rather than diagnosing a patient with VTS, a provider may instead classify the event as a “blighted ovum,” leading to further misclassification and misunderstanding of VTS’s clinical implications. VTS also serves as a biological and clinical confounder in fetal development research, with evidence suggesting that male VTS survivors may carry biological vulnerabilities that impact infant survival rates [9]. This finding suggested that VTS has been an overlooked factor in studies linking fetal size to infant mortality, and suggests that selection against frail male twins in utero may partially explain fluctuations in neonatal mortality rates.

This systematic literature review aims to assess clinical and psychosocial gaps in the diagnosis, management, and communication of VTS. Specifically, the review addresses the following research questions:

1. What are the physical and psychological risks associated with VTS for mothers and surviving multiples?
2. How do healthcare providers communicate with patients regarding VTS, and what barriers exist to effective communication?
3. How do international clinical guidelines vary in their treatment of VTS, and what opportunities exist for global harmonization or improvement?

## 2. Materials and Methods

This hybrid review combines narrative and systematic features to assess clinical and psychosocial gaps in the diagnosis, management, and communication of VTS. This format was selected to accommodate the limited availability of high-quality quantitative data in some domains while still applying systematic principles for literature selection and analysis. To explore these questions, we conducted a systematic literature review using key search terms such as “vanishing twin syndrome,” “patient-provider communicat*,” and “bereave* care.” Studies were selected based on their relevance to patient experiences, international clinical guidance, and reported physical and psychological outcomes associated with VTS. Medline/PubMed yielded 83 results on VTS. CINAHL retrieved 18 sources with the same term. Similarly, PsycINFO offered 4 sources. EBM Reviews offered 5 sources on VTS, whereas Scopus had 113. Due to the limited landscape of research surrounding VTS, no exclusion criteria were utilized in search queries. However, a number of sources older than ten years were excluded from this review due to outdated information. The evidence obtained comprised systematic reviews and meta-analyses, randomized controlled trials, cohort studies, and qualitative studies.

## 3. Results

In addition to challenges with the terminology used to describe the type of fetal loss, providers often struggle with how to communicate fetal loss effectively in both singleton and multiple pregnancies throughout gestation as well as at birth, leaving patients uninformed and unsupported [10,11]. Providers may opt not to inform their VTS patients that they have experienced a loss out of a desire to focus more on the positive aspects of the circumstances [12]. Poor communication regarding VTS is often not intentional or done out of malice; rather, the ways in which experienced physicians define and value life may impact what providers deem medically necessary to inform patients about—especially when at least one healthy embryo remains intact and/or when the loss occurs early on (e.g., before the detection of a viable heartbeat) [12]. However, withholding such information may lead to confusion or emotional distress. Parents who remain unaware of a loss may struggle to make sense of their own physical or emotional symptoms, such as unexplained grief, hormonal shifts, or abnormal screening results, thereby increasing anxiety. In contrast, several studies have demonstrated that patients who are better informed tend to experience lower levels of stress and anxiety, likely due to a clearer understanding of their circumstances and care options [13,14]. One study further indicated that well-informed patients tend to harbor more hope—often in the form of optimism for the healthy development of the surviving twin and confidence in their ability to cope with the pregnancy ahead [15].

### 3.1. Psychological and Legal Considerations

One study found that bereaved mothers of multiples generally reported significantly higher scores on the Perinatal Grief Scale compared to bereaved mothers of singletons, attributing this difference partially to the lack of recognition and validation from others [16]. An additional complication may arise from inconsistencies across institutions, states, and countries regarding the classification and management of fetal remains versus medical waste [17,18]. In the United States, federal regulations define a fetus as “the product of conception from implantation until delivery,” with “delivery” meaning complete separation of the fetus from the pregnant individual [19]. Additionally, a “dead fetus” is federally defined as one exhibiting no heartbeat, spontaneous respiratory activity, voluntary muscle movement, or umbilical cord pulsation [19]. However, national and state-level laws vary widely concerning handling, testing, and memorialization requirements for fetal remains. For example, Indiana enacted legislation (HEA 1337) requiring that fetal remains from abortions be either buried or cremated, treating them similarly to human remains [20]. In contrast, New York State law requires a burial permit and disposition of fetal remains only if the spontaneous abortion occurs after 20 weeks of gestation. Prior to this gestational age, there is no legal requirement for the mother to be informed of or involved in the disposal process [21]. This approach allows medical facilities to handle fetal remains from earlier gestational losses as medical waste, without imposing additional obligations on healthcare providers or patients. The international inconsistencies in the governance and terminology surrounding embryonic or fetal remains in England and other nations illustrate tensions between viewing them as medical waste, pregnancy remains, infant remains, or human corpse remains [22]. Furthermore, hospitals affiliated with distinct religious traditions (e.g., Catholic versus Adventist) often follow differing ethical guidelines when defining “life,” potentially further complicating decisions about disposal or memorialization of fetal tissues. This complexity is especially pronounced in cases of partial fetal resorption associated with VTS, where fetal remains may still be physically attached to the placenta of the surviving multiple(s).

### 3.2. Clinical and Developmental Complications

The clinical and developmental outcomes associated with VTS vary in their level of empirical support, with some complications well-established and others remaining controversial or poorly understood, as depicted in Table 1. Among the most consistent findings are placental pathologies (e.g., velamentous cord insertion, abnormal placental morphology, and placental weights below the 10th percentile), which are reported across multiple studies and are particularly associated with monochorionic pregnancies [4,23]. Conversely, associations between VTS and congenital anomalies, such as spina bifida or other fetal malformations, are largely based on limited case reports rather than robust cohort studies. Nevertheless, some studies have reported an increased prevalence of congenital anomalies, including spina bifida and other malformations, in surviving twins following early loss of a conceptus [24,25]. Additionally, the risk of cerebral palsy has been found to be higher among surviving twins after the intrauterine demise of a co-twin, further suggesting potential developmental vulnerabilities associated with VTS [26]. Reports of fetal malformations such as conjoined twins or partial resorption anomalies remain biologically plausible in early gestation but are rare and difficult to quantify due to limitations in detection and documentation.

#### 3.2.1. Fetal and Neonatal Complications

Survivors of VTS may be at increased risk for low birth weight and low Apgar scores; however, these findings are difficult to disentangle from baseline risks associated with multifetal gestations, particularly in the context of preterm birth or late gestational loss [27,28,29]. Other reported complications, such as intrauterine growth restriction (IUGR), gestational diabetes, placental abruption, placental sequelae (e.g., infarcts, hematomas, or fibrosis in the region where the fetus was lost), cervical insufficiency, chronic hypertension, and premature rupture of membranes (PROM), are inconsistently reported and often confounded by chorionicity, timing of loss, or use of ART [23,29,30,31]. In cases of VTS where remains fuse with the surviving co-twin(s) or their placenta or persist throughout gestation, there may be a diversion of resources that may elicit risks depicted in Table 1, including but not limited to neurodevelopmental impairment and fetal growth restriction [32,33]. Vaginal bleeding, while commonly observed in some VTS cases, is a nonspecific symptom that frequently occurs in early pregnancy and may be linked to a variety of unrelated causes, such as implantation, subchorionic hematoma, or early miscarriage. Although some studies report increased rates of bleeding in VTS, it is unclear whether this is a VTS-specific risk or reflective of broader multifetal or ART-related vulnerabilities [23,29]. Therefore, its association with VTS remains controversial and requires further controlled investigation.

#### 3.2.2. Mental Health Challenges

Psychologically, mothers experiencing VTS may suffer prolonged emotional distress from the time of loss through the postpartum period, underscoring the need for trauma-informed counseling and emotional support [28,34,35,36]. Similarly, surviving offspring—particularly monozygotic twins—have demonstrated increased risk of mood disorders and other emotional or behavioral challenges, although the evidence remains mixed due to variations in methodology and challenges controlling for confounding variables [4,37].

#### 3.2.3. Epigenetic and Developmental Disruption

Emerging research suggests that VTS may act as a biological confounder in fetal development. Epigenetic consequences of VTS (e.g., changes in DNA methylation and histone modification) have been proposed and warrant further investigation as they may impact gene expression in the surviving twin [38,39,40]. However, these outcomes are likely underreported due to the need for long-term testing and testing of multiple tissues, which is not routinely performed. Epigenetic changes following the loss of a multiple(s) can be considered pathological when they disrupt normal gene expression patterns in the surviving multiple(s), potentially affecting neurodevelopment, stress regulation, or long-term health. Such changes may have multigenerational implications. Moreover, changes may be triggered by intrauterine stress, vascular events, or the absorption of cellular material from the demised twin. As shown in Table 1, future research should aim to distinguish VTS-specific outcomes from those associated with general twin pregnancies while prioritizing long-term follow-up and stratification by gestational timing, chorionicity, and ART exposure.

**Table 1:** Risks linked to vanishing twin syndrome: Strength of evidence and confounders across studies.

Risk	Status in the Literature	Gestational Dependency	Primary Citations	Potential Confounders
Placental pathologies (e.g., small placentas, infarcts)	Well-established across studies	Across all gestational ages	[4,23,30]	Chorionicity, vascular events (e.g., twin-to-twin transfusion syndrome [TTTS]), chromosomal aneuploidies, SARS-CoV-2 and other pathogens
Maternal psychological distress	Well-established across studies	Not gestation-specific; linked to diagnostic experience	[34,35]	Healthcare interaction, cultural stigma
IUGR	Inconsistent results across studies	More likely in second- and third-trimester losses	[23,29]	Chorionicity, timing of demise
Vaginal bleeding	Inconsistent results across studies	Often in first trimester or with late loss	[23,29]	Common in early pregnancy, nonspecific symptom
Placental abruption	Inconsistent results across studies	Mid-to-late gestation	[29]	Vascular etiology, placental trauma
Cervical insufficiency	Inconsistent results across studies	Later gestation	[29]	Infection, uterine anomalies
Hypertension	Inconsistent results across studies	Later gestation	[29,31]	ART use, pre-existing maternal conditions
PROM	Inconsistent results across studies	Typically third trimester	[29]	Infection, chorionicity
Low birth weight	Inconsistent results across studies	More likely if demise occurs after 12–14 weeks	[27,29]	ART, chorionicity, gestational age at loss
Low Apgar scores	Inconsistent results across studies	More likely with late fetal demise	[28,29]	Gestational age at loss, co-occurring pathologies
Fetal malformations (e.g., spina bifida)	Inconsistent results across studies	Possibly embryonic period (<8–10 weeks)	[24,25,26,41]	Folate status, teratogens
Long-term stress, mood disorders in survivors	Inconsistent results across studies	All stages	[4,37]	Chorionicity, psychological history, zygosity
Chimerism in survivors	Rare, possibly underreported	More likely with early placental fusion (incl. fused DZ placentas)	[39,40]	Testing limitations, twin DNA admixture
Epigenetic effects (e.g., methylation changes) in survivors	Theoretical/emerging evidence	Inter-/multi-generational relevance suggested	[38]	Baseline variability, maternal exposures
Neurodevelopmental impairment in survivors	Well-established in monochorionic twin losses; unclear in early VTS	Most significant in second- to third-trimester losses in monochorionic twins	[30,42]	Chorionicity, timing of demise

Legend. Status in the literature: Well-established across studies: Findings consistently reported across multiple, high-quality studies with replication in different populations. Inconsistent results across studies: Evidence varies between studies, likely influenced by sample size, study design, chorionicity, gestational timing of loss, or other confounding factors. Rare, likely underreported: Observed primarily in isolated case reports or small cohorts; lack systematic investigation. Theoretical/emerging evidence: Proposed based on biological plausibility, case studies, or preliminary data; not yet validated through large-scale empirical studies.

#### 3.2.4. Genetic Anomalies and VTS Across Twinning Models

Although rare, chimerism may result from multiple pregnancies [43]. It typically arises from the fusion of two genetically distinct zygotes or the resorption of deceased fetal material into a co-developing fetus—processes that are more likely in dizygotic twin gestations. Thus, the likelihood and impact of chimerism vary with chorionicity, being more plausible in dichorionic pregnancies, and, to a lesser extent, amnionicity and gestational timing [1]. In contrast, monozygotic twins share nearly identical genetic material, making chimerism extremely rare in such cases. Chimerism may be detected by certain genetic testing modalities, such as chromosomal microarray (CMA) with SNP probes or genome sequencing, though some cases can go unnoticed. Most findings are incidental and clinically insignificant unless an abnormal cell line is involved. However, chimerism can affect histocompatibility and genetic testing interpretation and, in rare instances, has resulted in parentage discrepancies due to tissue-specific genetic variation [40]. Besides chimerism, fraternal dichorionic/diamniotic (DCDA) twins often present the lowest risks for adverse outcomes compared to multiples that share the same sac and/or placenta [25,44]. This, therefore, highlights the need for both providers and patients to be better informed about fetal loss and the specific characteristics of multiple types (e.g., chorionicity and amnionicity).

While VTS and conditions such as teratomas, parasitic twins, and fetus in fetu are typically understood as distinct entities, some researchers have hypothesized embryologic overlap. Rare case reports suggest that remnants of a resorbed twin in early gestation might contribute to the development of complex masses, including teratomas or malformed parasitic structures [45,46,47]. Although teratomas are most often attributed to pluripotent germ cell anomalies, more speculative theories point to early twinning errors or the incorporation of embryonic tissue during resorption as possible contributing factors [48,49]. Fetus in fetu, often considered a severe form of parasitic twinning, has also been proposed to overlap histologically with teratomas due to similar tissue composition and imaging characteristics [47]. However, these connections remain theoretical, with most reports lacking molecular evidence of a preceding twin demise. Moreover, if such developmental disruptions occur pre-implantation, they will fall outside the diagnostic scope of VTS, which requires post-implantation evidence of a vanished co-twin [1]. Thus, while biologically plausible, a causal link between clinically recognized VTS and parasitic outcomes remains unproven and should be interpreted with caution.

Mirror-image twinning (i.e., with mirrored traits such as handedness, dermatoglyphics, or craniofacial features) has been described in the literature as part of the spectrum of atypical outcomes of twinning, alongside phenomena such as parasitic twins and fetuses in fetu [49]. Beyond major visceral differences, mirror-imaging has also been documented in external phenotypes such as dental crown morphology [50]. Rare instances of situs inversus totalis (i.e., visceral organs are fully reversed) have been observed in monozygotic twins, including cases where only one co-twin exhibited reversal [51]. Additionally, reports of heterotaxy syndrome with mirror-image anatomical anomalies in identical twins support the association between twinning events and laterality disruptions [52]. Mirror-image twinning is overall estimated to occur in approximately 25% of monozygotic twin pairs and is thought to arise when embryonic splitting occurs relatively late in development, potentially after left–right patterning cues have been established. According to the traditional fission model, dizygotic (fraternal) twins originate from the fertilization of two separate eggs, while monozygotic (identical) twins result from the postzygotic division of a single fertilized egg [53]. Given that vanishing twin syndrome (VTS) represents an early disruption in embryonic development, it is biologically plausible that the loss of a co-twin could further perturb left–right axis signaling during critical windows of organogenesis. Such perturbations may increase the likelihood of mirror-image outcomes or laterality anomalies in surviving twins, although direct empirical studies confirming this relationship remain limited. As previously mentioned, VTS can occur at various developmental stages depending on the mechanism of twinning and the timing of embryonic events. According to the traditional fission model, dizygotic (fraternal) twins originate from the fertilization of two separate eggs, while monozygotic (identical) twins result from the postzygotic division of a single fertilized egg [54]. The timing of this division determines the chorionicity and amnionicity of the pregnancy as follows: early splits (days 1–3) result in dichorionic diamniotic (DCDA) twins, mid-stage splits (days 4–8) produce monochorionic diamniotic (MCDA) twins, and late splits (days 8–13) lead to monochorionic monoamniotic (MCMA) twins. If the split occurs after day 13, the result may be conjoined twins. Each of these scenarios carries a different risk profile for VTS. For example, MCDA and MCMA twins, who share a placenta or both placenta and amniotic sac, respectively, are more vulnerable to complications like twin-to-twin transfusion syndrome (TTTS), selective intrauterine growth restriction (IUGR), or hemodynamic instability if one twin is lost.

In contrast, Herranz’s alternative model proposes that monozygotic twinning results from the first cleavage of the zygote, and subsequent membrane fusion within the zona pellucida—not timing of splitting—determines chorionicity [54]. This model suggests VTS may result from early asymmetries or failure in membrane separation, particularly in fused trophectoderm or inner cell masses. Atypical twinning processes, such as chimeric twinning, polar body fertilization, or superfetation, offer further mechanisms for early embryonic loss. Each of these introduces unique pathways for VTS to occur, including the fusion of two zygotes, differential survival of polar body-derived embryos, or spatial competition between asynchronously developing embryos. The timing of fetal loss also influences VTS outcomes as follows: early losses (before 9 weeks) are often asymptomatic and undetected without ultrasound, whereas later losses (after 10 weeks) can result in identifiable manifestations such as fetus papyraceus or neurological risks to the surviving twin. Overall, VTS is a multifactorial event that intersects with both classical and evolving models of human embryonic development. The variances in VTS across these models are depicted in Table 2 and Table 3 below.

#### 3.2.5. VTS and Prenatal Testing

VTS and other types of multiple loss may impact screening results, including maternal serum screening (MSS). MSS measures hormones and proteins such as human chorionic gonadotropin (hCG), pregnancy-associated plasma protein-A (PAPP-A), and alpha-fetoprotein (AFP) in maternal blood, which may be abnormally elevated or suppressed due to the presence or loss of a second fetus. In VTS cases, particularly following early loss, trophoblastic tissue from the demised embryo may remain metabolically active, producing hormones like hCG and sustaining limited vascular activity. This residual trophoblastic activity can result in persistently abnormal serum marker levels. While PAPP-A and AFP levels tend to normalize over time, hCG and inhibin A may remain elevated for more than four weeks after fetal demise, complicating the interpretation of first- and second-trimester screening results [55]. Although nuchal translucency (NT) itself may not be directly altered, its interpretation in conjunction with these serum markers can yield inaccurate risk estimates. For this reason, MSS is generally not recommended in pregnancies affected by VTS due to the high likelihood of false-positive or false-negative results [55]. Pregnancies complicated by a vanishing twin had significantly altered first- and second-trimester maternal serum marker levels compared to singleton pregnancies, including elevated hCG and inhibin A and decreased PAPP-A, further supporting concerns regarding the interpretation of MSS results in the context of VTS [41].

Identification of VTS is also critical for interpreting the results of prenatal cfDNA screening for the ongoing pregnancy. Approximately 50% of all early pregnancy losses may result from chromosomal abnormalities [56], and DNA from a demised co-twin may continue to be detected in cfDNA for up to 15 weeks after the loss [57]. Therefore, vanishing twins are a common source of false positives in prenatal cfDNA screening [4,58]. It is recommended that positive prenatal cfDNA screening results be confirmed by diagnostic testing, which involves a cytogenetic assessment of DNA collected from chorionic villus sampling (CVS) or an amniocentesis. Given that VTS impacts the accuracy of cfDNA screening, a history of VTS for the ongoing pregnancy warrants additional discussion with a genetic counselor regarding the likelihood of a true positive upon diagnostic testing. VTS must also be considered when the pregnant individual receives a low-risk prenatal cfDNA result, as other discrepancies can occur, including an incorrect sex call [59]. Prenatal cfDNA screening results may represent the status of the ongoing pregnancy, the co-twin demise, the placenta, a maternal condition, a combination of these factors, or a false positive. As a result, this screening assay cannot diagnose the genetic status of the pregnancy loss. This information may be important for the health of the pregnant individual. For example, some partial hydatidiform moles (PHM; resulting from the fertilization of a normal egg by two or more sperm) and complete hydatidiform moles (CHM; resulting from the fertilization of an enucleated egg by one or two sperm) can cause gestational trophoblastic disease, which rarely for PHM and more frequently for CHM can develop into gestational trophoblastic neoplasia [60,61]. In these cases, serial testing of hCG levels after removal of molar tissue from the uterus is important to monitor for risks of transforming into a malignant condition [60].

Genetic diagnostic testing of the loss may also be desired for a psychological benefit to parents and to identify if there is a significant risk of recurrence for a given genetic abnormality in future pregnancies [62]. Diagnostic testing can be performed on products of conception after delivery of the ongoing pregnancy, which can often be identified by examination of the placenta by a pathologist. Referral to a genetic counselor is warranted given the complexity of this testing. For example, cytogenetic assessment of the tissue should involve CMA and not karyotype, which requires successful tissue culture from fresh living tissue that would not be available for older deceased remains. If information about triploidy is desired (for example, diandric triploidy, which results in PHM), a CMA should include SNP probes. Results should be interpreted with caution, as they may be confounded by maternal cell contamination and/or the surviving co-twin.

### 3.3. Existing Guidelines and Their Limitations

Establishing guidelines that serve pregnancies where there is a loss of one or more fetuses can be challenging, as they would ideally necessitate the involvement of professionals who comprise the complex system of care coordination [36]. For example, guidelines may include the need for nuanced interpretation of serum and prenatal cfDNA screening results, as well as patient options or resources for fetal remains disposal or diagnostic genetic testing, memorialization, bereavement, and counseling. This lack of information is likely to be due in part to poor translation of research from the bench to the bedside for both providers and institutions [63]. Improving comprehensive care for pregnancies affected by VTS involves addressing several complex challenges, such as the following:

- Underdiagnosis and Limited Literature: VTS is often underdiagnosed, especially in naturally conceived pregnancies, leading to a scarcity of comprehensive studies and literature on the condition. This lack of data hampers the development of evidence-based guidelines and care.
- Perceived Importance by Providers: As previously cited, some healthcare providers may not fully recognize the physical and emotional significance of VTS depicted in Table 1, resulting in inconsistent management approaches and a lack of standardized care protocols.
- Composition and Availability of Care Teams: Effective management of VTS requires a multidisciplinary team, including obstetricians, genetic counselors, mental health professionals, and bereavement counselors. However, access to such professionals is not uniform across regions. For instance, the Southern United States has a notably low number of genetic counselors, averaging 0.69 per 100,000 residents, with many concentrated in urban areas. This disparity limits access for patients in rural communities [64]. Telemedicine and digital health tools may help address these gaps by enhancing access to remote mental health services, virtual genetic counseling, and app-based pregnancy monitoring. Such interventions can offer timely emotional support and facilitate care coordination, particularly in the aftermath of early pregnancy loss when in-person follow-up may be difficult to access.
- Insurance: Coverage for most genetic services remains inconsistent, leading to potential out-of-pocket expenses for VTS patients [65,66]. This trend disproportionately affects less privileged women and exacerbates existing healthcare disparities [67].

Organizations such as the American College of Obstetricians and Gynecologists (ACOG), the Royal College of Obstetricians and Gynaecologists (RCOG), the International Society for Prenatal Diagnosis (ISPD), the Society for Maternal-Fetal Medicine (SMFM), and the International Society of Ultrasound in Obstetrics and Gynecology (ISUOG) have offered guidance on multiple pregnancies more broadly, but the focus on VTS is variable.

ISUOG provides comprehensive guidance on early pregnancy ultrasound, with emphasis on the determination of chorionicity and amnionicity. In its 2025 update, ISUOG references the “vanishing twin phenomenon” during chorionicity assessment and acknowledges that earlier gestational losses may still allow for retrospective chorionicity determination [30]. However, its guidance does not extend into the clinical implications of VTS, such as placental sequelae (e.g., infarcts, hematomas, fibrosis), or psychosocial care for families affected by early loss in multiple pregnancies.

ISPD similarly includes detailed protocols for prenatal screening and diagnosis, particularly in multifetal pregnancies, but lacks VTS-specific recommendations for interpreting cfDNA results or managing residual confounders from a demised co-twin. Although ISPD addresses limitations in cfDNA interpretation following fetal demise in general, it lacks more targeted guidance for VTS-affected pregnancies.

ACOG addresses VTS primarily in the context of prenatal screening. Specifically, it notes that in multifetal gestations where a fetal demise, vanishing twin, or anomaly is identified in one fetus, there is a significant risk of inaccurate test results if serum-based or prenatal cfDNA screening are used. In such cases, diagnostic testing should be offered [68]. Beyond these considerations, ACOG’s guidelines do not provide detailed recommendations for the broader clinical management of VTS, such as monitoring protocols for the surviving fetus or tailored counseling for affected parents. However, in the absence of formal guidance, expert consensus and case-based practices often include serial ultrasounds to monitor fetal growth and amniotic fluid volume, Doppler studies for assessing blood flow abnormalities (particularly in monochorionic twins), and postnatal placental evaluation [36,69].

SMFM does not offer a dedicated guideline for the management of VTS. However, it does reference VTS in clinical and coding guidance relevant to early pregnancy assessment and prenatal care. SMFM emphasizes the importance of early ultrasound to establish gestational age, confirm viability, determine the number of fetuses, and identify conditions like a vanishing twin or empty gestational sac. This is crucial for accurate counseling and understanding fetal risks [70]. It also provides coding recommendations for appropriately documenting cases involving a vanishing twin, which is essential for ongoing pregnancy management and insurance coverage [71].

The National Institute for Health and Care Excellence (NICE) in the United Kingdom provides comprehensive maternal health guidelines, which include antenatal management of multiple pregnancies through fetal monitoring, counseling support for parents, and screening for complications like TTTS, Twin Anemia Polycythemia Sequence (TAPS), selective fetal growth restriction (sFGR), and twin reversed arterial perfusion (TRAP) sequence [36,72]. NICE quality standard QS46 recommends determining chorionicity and amnionicity early in pregnancy, between 11 weeks and 2 days to 14 weeks and 1 day or as soon as possible, which has contributed to reducing twin stillbirths; however, individuals experiencing VTS were excluded from related analyses [73,74]. Guidelines in France, from the Collège National des Gynécologues et Obstétriciens Français (CNGOF), discuss risks associated with intrauterine fetal demise but do not specifically mention VTS [75].

Beyond the countries listed above, there are limited clinical guidelines specifically addressing VTS, notably across African and Nordic countries, Australia, China, Russia, Latin America, and the International Federation of Gynecology and Obstetrics (FIGO). Despite having the highest twinning rate of any continent and with many cultures having customs surrounding the birth and death of twins in pregnancy and postpartum (e.g., Yoruba), a number of African nations (Rwanda, Mozambique, and Tanzania) are in need of more detailed guidelines on the management of twin pregnancies [43,76,77,78]. One exception is South Africa, where the South African Society of Ultrasound in Obstetrics and Gynaecology (SASUOG) has established guidelines on the management of twin pregnancies that mention potential impacts of VTS on prenatal cfDNA screening [79]. In Nordic countries (Sweden, Denmark, Norway, Finland, and Iceland), guidelines developed by the Nordic Federation of Societies of Obstetrics and Gynecology (NFOG) broadly address various obstetric scenarios, including twin deliveries, but do not specifically include VTS [80].

The Royal Australian and New Zealand College of Obstetricians (RANZCOG) also lacks formal national guidelines explicitly addressing VTS, though general obstetric guidelines address the management of multiple pregnancies (e.g., TTTS) and fetal demise in monochorionic twin pregnancies [81]. Despite the lack of guidelines in Australia, major companies like Huggies Australia, for example, provide online information on VTS, noting that while the surviving twin is usually unaffected, there is an increased risk of preterm birth and low birth weight [82].

The Chinese Society of Perinatal Medicine (CSPM) and the Chinese Society of Obstetrics and Gynecology (CSOG) similarly have not established national guidelines for managing VTS [83,84]. Studies from China have highlighted the association between VTS and adverse perinatal outcomes, such as preterm birth and low birth weight, but these findings have not translated into formal clinical guidelines [83]. In South Korea, the Korean Society of Maternal-Fetal Medicine (KSMFM) has developed guidelines focusing on maternal serum screening and prenatal cfDNA screening, but these lack specific recommendations for managing VTS care [55].

In contrast, Canada’s national guidelines, provided by the Society of Obstetricians and Gynaecologists of Canada (SOGC), acknowledge VTS explicitly, recognizing associated risks such as fetal structural anomalies, growth restrictions, and preterm birth [36,85]. 

Given the emotional and medical complexities involved with VTS, comprehensive guidelines are needed globally to guide healthcare providers in communication, emotional and psychological care, genetic testing, and medical management following fetal loss. This may include medical management strategies such as enhanced ultrasound surveillance for the surviving fetus, placental pathology evaluation, postpartum follow-up for maternal complications, and referrals to maternal–fetal medicine specialists when warranted. The absence of formal standards can lead to significant psychological impacts such as unresolved grief, anxiety, and depression among affected families, potentially influencing parenting and intergenerational health through mechanisms such as trauma-induced epigenetic modifications [37,38]. Thus, establishing detailed and inclusive VTS-specific guidelines is essential to effectively address these significant public health implications. Table 4 summarizes key guideline findings.

### 3.4. Recommendations for Future Guidelines

Future iterations of the ISUOG Practice Guidelines, along with those of other professional societies such as ISPD, ACOG, and SMFM, could be enhanced by expanding their guidance on VTS. This is particularly important following early first-trimester loss, which remains the most common period for this phenomenon. Given ISUOG’s role as the leading authority on obstetric ultrasound standards and ISPD’s leadership in prenatal genetic diagnostics, both organizations are well-positioned to incorporate VTS-specific clinical recommendations into updated protocols. Additionally, future guidelines should integrate interdisciplinary approaches to care coordination and ensure that both patients and providers have access to standardized protocols that address bereavement support and informed decision-making. In the event of a loss of multiples, the network of care may become even more intricate than standalone obstetric care. This is especially problematic in health systems where the burden of care is on the patient, as care can be extremely complex to navigate. Addressing these gaps through well-defined and inclusive policies will be essential in improving outcomes for individuals affected by VTS, particularly in cases occurring beyond the first trimester, where adverse outcomes in survivors and mothers have been more frequently reported.

To be effective, future guidelines should be explicit, practical, and applicable across different clinical contexts. Providers need clear protocols that address diagnostic criteria, disclosure practices, timing and interpretation of genetic testing, and bereavement support pathways. While VTS, TTTS, TAPS, sFGR, and TRAP are distinct conditions that warrant equally distinct diagnostic and care criteria, it is important for guidelines and screening protocols to account for how they can be related, as many of these conditions can lead to the loss of one or both twins [86]. These conditions are more likely to occur in monochorionic twins due to vascular connections in the shared placenta (i.e., placental anastomoses) [86]. While guidelines should emphasize the elevated risks in monochorionic twins, they must also ensure that dichorionic twins are not overlooked, as they too carry risks. Both chorionic types warrant appropriately stratified care.

Key questions remain unanswered: Should the pregnancy be managed as a twin or singleton after one fetus demises? What are the implications for maternal health, and to what degree are the surviving fetuses at neurological or hemodynamic risk due to shared placental features? Given that many of the risks associated with VTS, such as neurodevelopmental outcomes or maternal complications, are primarily documented following second- or third-trimester losses, future guidelines should clarify that current care recommendations (e.g., fetal growth monitoring, placental pathology, and mental health referrals) may be especially valuable for later losses. Evidence is still limited regarding first-trimester losses, especially in naturally conceived pregnancies, highlighting the need for tailored guidance based on timing and clinical context. It is also important to acknowledge that many of the associated risks and conditions are not specific to VTS, but reflect correlations where causality may be difficult to discern due to similarities with typical pregnancy symptoms or overlapping comorbidities.

### 3.5. Enhanced Monitoring Recommendations

For pregnant individuals:

- Early and Frequent Prenatal Monitoring: Initiate early prenatal visits to monitor fetal viability, assess chorionicity, and detect potential complications. When feasible, early referral to a maternal-fetal medicine (MFM) specialist is recommended.
- Chorionicity Disclosure and Patient Education: Inform patients about chorionicity as early as possible. SNP-based cfDNA screening may aid in zygosity determination [87], though results should be interpreted cautiously in VTS cases.
- Genetic Counseling and Prenatal Screening Adjustments: Consider confirmatory diagnostic testing like amniocentesis or CVS. Interpret prenatal cfDNA and MSS results carefully and involve genetic counselors to assist in evaluation where necessary, based on case circumstances. Alternative strategies such as nuchal translucency in combination with maternal age may be useful [88]. Diagnostic confirmation should be prioritized when indicated [68].
- Psychological and Bereavement Support: Offer individualized support, including referrals to counseling, charities, and mental health services.
- Resources for Handling Fetal Remains: Provide resources on funeral homes and legal definitions of remains. Consider diagnostic/pathological testing of remains when appropriate. Bereavement resources such as the Butterfly Project have been shown to be particularly helpful in hospital neonatal intensive care units (NICUs) [89].
- Nutritional and Lifestyle Guidance: Provide tailored nutritional counseling to support maternal health and fetal development, particularly in ongoing multiple pregnancies affected by VTS. In cases where fetal resorption or placental abnormalities are suspected, nutritional support may help mitigate maternal inflammation or promote optimal growth for the surviving fetus. Emphasis should be placed on protein, folic acid, iron, and micronutrients essential for tissue repair and hematologic stability [90].
- Postpartum Monitoring: Screen for delayed or secondary complications in mothers who experience VTS (e.g., hypertension and autoimmune responses). Emotional stress from unresolved grief may also contribute to somatic symptoms and should be monitored in coordination with mental health support services both during pregnancy and postpartum.

For Surviving Multiples:

- NICU Readiness: Ensure access to an appropriate level NICU, especially for preterm or low birth weight infants. The NICU should ideally offer bereavement resources tailored to families experiencing the loss of a multiple (e.g., the Butterfly Project).
- Developmental Assessments: Screen early for behavioral and cognitive issues. Monitor for congenital malformations more commonly seen after later gestational losses of a multiple(s).
- Routine Physical Checks: Include screening for low Apgar scores and anomalies potentially linked to vascular compromise.
- Mental Health Monitoring: Conduct long-term psychological evaluations, particularly in monozygotic survivors.
- Genetic Testing: Consider in rare cases of suspected chimerism.
- Parental Education and Support: Educate parents on possible developmental or emotional impacts.
- Multidisciplinary Care Approach: Encourage coordination among neonatologists, geneticists, psychologists, and pediatricians.

### 3.6. Terminology and Survivor Identity

The term “vanishing twin syndrome” is widely recognized, but it is medically imprecise and potentially harmful. The label suggests a process that is both final and uneventful, which may not reflect the clinical or emotional reality for patients. Instances where VTS is referred to as the “vanishing twin phenomenon” further undermine its recognition as a diagnosable obstetric event [12]. This vague characterization negatively affects diagnostic accuracy, care planning, and empathetic patient communication.

Moreover, the use of the term *twin* (singular) or *twins* (plural) narrows the perceived scope of the condition, despite the fact that VTS can occur in pregnancies involving triplets or higher-order multiples. The current terminology excludes these cases by default, limiting understanding and perpetuating gaps in clinical documentation and support. In cases where fetal remains are not fully resorbed or expelled, and thus do not “vanish,” what has occurred is often a form of a missed abortion [91]. Future research should explore how frequently terms like “missed abortion” or “blighted ovum” are used in clinical settings to describe VTS, and how such language may influence outcomes and follow-up care.

Whether it manifests as a blighted ovum, resorbed fetal tissue, or fetus papyraceous, VTS aligns more accurately with existing diagnostic criteria such as a “missed abortion” or “pregnancy loss.” Framing VTS within the broader spectrum of miscarriage may improve empathy and clinical transparency, facilitate appropriate documentation, and expand access to psychosocial and bereavement support. Terminology that minimizes or oversimplifies fetal loss can shape how healthcare professionals communicate with patients, how patients interpret their experiences, and how systems prioritize care and policy development [10]. Patients navigating this form of loss may be left with little clarity and even less support if the language used implies that the event was minor or self-resolving [34]. As a result of these linguistic complexities and inadequacies, existing terminology may contribute to a limited understanding of the condition’s full range of impact, affecting both patient and provider decision-making. Alternative terms such as “spontaneous co-twin demise” (in cases of twins), “intrauterine fetal demise of one twin [or triplet, etc.],” “[single/twin/triplet] fetal death in the [X] trimester,” or “intrauterine demise in a multiple gestation” offer more accurate clinical descriptions [92,93,94]. “Vanishing” may imply an erasure or insignificance of the lost embryo, fetus, or empty gestational sac. “Vanishing twin syndrome” has been critiqued for sounding clinical, impersonal, and dismissive [12]. The term implies that the loss is simple or uneventful, which is often not the case emotionally or physically [12,34].

One term proposed in the literature for the survivors of multifetal loss that has not been frequently used includes “womb twin,” which centers on the lived experience of the surviving twin, recognizing the enduring emotional and psychological impact of the prenatal loss [12]. “Womb twin” may also be appropriate to describe the survivors and hosts of conjoined and parasitic twins, respectively. Moreover, “womb twin” validates that a co-twin once existed and may continue to influence the survivor’s identity, health, and well-being. These alternative terms are less ambiguous and better reflect the biological, emotional, and psychological complexities most often involved with VTS, as well as other types of multifetal loss, for both pregnant individuals and survivors.

## 4. Limitations

There is marked heterogeneity in study designs, cohort demographics, and clinical outcomes reported across the literature. Additionally, studies vary widely in how and when VTS is diagnosed, with many relying on retrospective ultrasound findings or incomplete chorionicity data, which may lead to misclassification. Limitations also include the small sample sizes of certain subgroups, limited follow-up duration to evaluate neurodevelopmental outcomes, and inconsistent reporting of zygosity, amnionicity, and chorionicity. These inconsistencies contribute to potential biases, including selection bias, recall bias, and publication bias, particularly in studies that rely on parental self-reporting or retrospective chart reviews. Furthermore, the lack of standardized criteria for diagnosing VTS and the potential for early losses to go undetected in natural pregnancies suggest underreporting and may skew prevalence data. These factors highlight the need for more rigorous, prospective, and stratified research to inform accurate risk assessments and clinical guidelines.

## 5. Discussion

Exploring the experiences of VTS patients and improving patient care in cases of embryonic, fetal, and infant death of multiples is a multifaceted endeavor. Despite existing gaps in both academic literature and policy guidelines regarding VTS, there is a clear need for effective, evidence-based management protocols. Moving forward, addressing these gaps requires further research not only on parental VTS experiences but also on the physical and psychological trajectories of surviving VTS children. Future studies should strive to understand the informational needs, emotional and physical support requirements, and long-term outcomes of affected families. A critical component of improving care involves re-evaluating the classification of VTS within clinical and institutional frameworks. Reclassifying VTS as a form of miscarriage—particularly in cases of early embryonic or fetal loss—would align diagnostic language with patient experiences, reduce documentation inconsistencies, and enable access to bereavement resources that are otherwise unavailable. Including this reclassification in clinical guidelines could validate the grief of affected families and encourage providers to offer appropriate support. Efforts should also focus on translating research findings into comprehensive patient-centered care models. Collaboration among patient advocates, healthcare providers, researchers, and policymakers is essential for developing standards that reflect the complexity of VTS and its impact on families. Strategies for assessing intervention quality may include well-powered studies that use robust statistical analyses to evaluate outcomes, enabling evidence-based changes to care protocols.

To advance clinical practice and policy development, future research must address issues of reproducibility and generalizability. Many reported associations—such as low birth weight, placental abruption and vaginal bleeding—have not been replicated consistently, likely due to heterogeneity in study design, cohort selection, and confounding factors like chorionicity, amnionicity, and ART use. Well-powered, prospective cohort studies are needed to disentangle outcomes specifically attributable to VTS from those associated with multifetal gestation more broadly. Incorporating stratification by gestational age at loss (e.g., first vs. later trimesters), chorionicity (e.g., monochorionic vs. dichorionic), and ART exposure into study design will enhance comparability across populations. Moreover, establishing multicenter registries to systematically document VTS diagnoses, prenatal screening results, fetal remains management, and long-term outcomes will improve data standardization and clinical benchmarking. The integration of biological samples—such as placental tissue, maternal serum, and cfDNA—into these registries can further support molecular investigations, including potential epigenetic markers or other biomarkers of VTS-related complications.

## 6. Conclusions

VTS presents complex clinical, psychological, and diagnostic challenges that remain under-recognized in current healthcare systems and international guidelines. Despite increased detection through early ultrasound and ART, standard care protocols for VTS are inconsistent and often fail to meet the emotional and medical needs of affected families. This review highlights the importance of early chorionicity assessment, improved communication strategies, and multidisciplinary support for both parents and surviving multiples. To optimize outcomes, future clinical guidelines must address diagnostic clarity, prenatal screening interpretation, survivor monitoring, and bereavement support when needed. Recognizing VTS as a meaningful form of pregnancy loss—and reclassifying it within miscarriage terminology—can enhance empathy, documentation, and care continuity, ultimately contributing to a more patient-centered standard of care.

## Figures and Tables

**Table 2:** VTS via the traditional model (fission-based monozygotic and dizygotic twinning).

Stage	Twinning Mechanism	Chorionicity/ Amnionicity	VTS Risk and Mechanism
Days 0–3 (Morula)	Dizygotic or monozygotic splitting	DCDA	VTS may occur independently for either twin. Loss likely goes unnoticed unless early ultrasound is performed.
Days 4–8 (Blastocyst)	Monozygotic splitting	MCDA	Shared placenta raises risk for TTTS, selective IUGR, and vascular complications after co-twin loss.
Days 8–13 (Implanted blastocyst)	Monozygotic splitting	MCMA	Shared amniotic sac introduces risks like cord entanglement; VTS may cause hemodynamic complications.
After day 13	Incomplete split → Conjoined twins	MCMA	VTS here may manifest as fetus papyraceus or lead to parasitic twins/fetus in fetu.

**Table 3:** VTS via Herranz’s Alternative Fusion-Based Model.

Stage	Mechanism	Outcome	VTS Risk and Mechanism
First cleavage (two-cell stage)	Two daughter cells each form an embryo	Potential DCDA	If embryonic membranes fuse, VTS might result from competition or failed ICM coordination.
Blastocyst stage	Two blastocysts fuse trophectoderm	MCDA or MCMA	Fusion increases vulnerability in shared placenta, raising VTS risk via perfusion mismatch.

**Table 4:** Current guidelines: What is addressed and what is still needed.

Organization	What Is Addressed	What Is Still Needed
**ISUOG**	Chorionicity and early loss recognition during ultrasound	VTS-specific care protocols (e.g., psychosocial care, placental pathology)
**ISPD**	Prenatal screening and diagnostics in multifetal pregnancies	VTS-specific cfDNA interpretation guidance
**ACOG**	Risks of inaccurate screening after fetal demise, recommendations for diagnostic testing	Guidance for monitoring of survivors, bereavement care, and VTS terminology
**SMFM**	VTS in early pregnancy ultrasound and coding guidance	Dedicated management guideline for VTS beyond administrative references
**NICE**	Antenatal management of multiples, determination of chorionicity	Inclusion of VTS outcomes and psychosocial support in twin-loss cases
**CNGOF**	General fetal demise guidance, not VTS-specific	Explicit inclusion of VTS cases and terminology refinement
**RANZCOG**	General multiple pregnancy and demise care, not VTS-specific	Formal VTS guidelines with care stratification by gestational timing
**SOGC**	Risks associated with VTS (e.g., anomalies, growth restriction, preterm birth)	Detailed management guidance and interdisciplinary care protocols
**SASUOG**	VTS impact on cfDNA screening	Expanded psychosocial, genetic counseling, and emotional care guidelines
**NFOG**	Twin delivery and obstetric guidance, not VTS-specific	Specific guidance for VTS recognition, terminology, and follow-up care
**CSPM and CSOG**	Extensive VTS research	Translation of research into national clinical recommendations
**KSMFM**	Maternal serum and cfDNA screening protocols without VTS-specific guidance	Inclusion of VTS in screening interpretation and psychological support

## Data Availability

The original contributions presented in this study are included in the article. Further inquiries can be directed to the corresponding author.
